# Blueberry Supplementation in Midlife for Dementia Risk Reduction

**DOI:** 10.3390/nu14081619

**Published:** 2022-04-13

**Authors:** Robert Krikorian, Matthew R. Skelton, Suzanne S. Summer, Marcelle D. Shidler, Patrick G. Sullivan

**Affiliations:** 1 Department of Psychiatry & Behavioral Neuroscience, University of Cincinnati Academic Health Center, Cincinnati, OH 45267, USA; shidlemd@ucmail.uc.edu; 2 Division of Neurology, Cincinnati Children’s Research Foundation, Cincinnati, OH 45229, USA; matthew.skelton@cchmc.org; 3 Bionutrition Core, Clinical Translational Research Center, Cincinnati Children’s Hospital Medical Center, Cincinnati, OH 45229, USA; suzanne.summer@cchmc.org; 4 Spinal Cord & Brain Injury Research Center, Chandler College of Medicine, University of Kentucky, Lexington, KY 40506, USA; patsullivan@uky.edu

**Keywords:** BMI, insulin resistance, cognition

## Abstract

Late-life dementia typically develops over a period of many years beginning in midlife. Prevalence of metabolic disturbance also accelerates in middle age and is a prominent risk factor for dementia. Preliminary studies indicate that blueberry supplementation can improve cognitive performance and influence metabolism and brain function and therefore may have a role in early intervention to prevent neurodegeneration. In a randomized controlled trial, we investigated the effects of daily blueberry supplementation in a middle-aged sample of insulin-resistant participants with elevated risk for future dementia. We enrolled overweight men and women, aged 50 to 65 years, with subjective cognitive decline (SCD) and performed pre- and post-intervention assessments of cognition and metabolism and exploratory measures of peripheral mitochondrial function. We observed improved performances for the blueberry group on measures of lexical access, *p* = 0.003, and memory interference, *p* = 0.04, and blueberry-treated participants reported reduced memory encoding difficulty in daily life activities, *p* = 0.03. The blueberry-treated group also exhibited correction of peripheral hyperinsulinemia, *p* = 0.04, and a modest trend for increased mitochondrial uncoupling, *p* = 0.11. The cognitive findings indicated improved executive ability in this middle-aged sample. In addition, the changes in metabolic and bioenergetic measures imply potential mechanistic factors associated with anthocyanin and proanthocyanidin actions. The demonstration of these benefits in middle-aged individuals with insulin resistance and SCD suggests that ongoing blueberry supplementation may contribute to protection against cognitive decline when implemented early in at-risk individuals.

## 1. Introduction

Nearly six million older adults live with dementia in the United States [1]. Alzheimer’s disease (AD) accounts for up to 80% of dementia cases, and it is projected that there will be as many as 14 million cases of AD by the year 2050 [1]. There is no treatment for dementia, and it is not clear when effective therapy might be developed. Accordingly, preventive approaches and mitigation of risk for cognitive decline represent the optimal means of coping with this public health challenge. Targeting modifiable risks, such as poor nutrition and related metabolic disturbance, are among the more prominent emerging preventive approaches [2,3].

Neurodegenerative changes associated with late-life AD and other dementing conditions begin many years before the appearance of functional decline. This preclinical period, beginning in midlife, represents an opportunity for early intervention by avoiding or treating health conditions that increase risk. Metabolic disturbance is implicated in most chronic diseases of aging including dementia [4]. Unfortunately, nearly 50% of middle-aged adults in the U.S. have insulin resistance [5], a condition that involves insulin receptor insensitivity and compensatory increase in insulin secretion. Thus, insulin resistance is a hyperinsulinemic state [6]. Peripheral hyperinsulinemia is associated with reduced insulin transport into the brain, and central hypoinsulinemia promotes neurodegeneration through reduction of neurotrophic factors; greater retention of amyloid beta, a pathological feature of AD; and through other mechanisms [7,8,9,10]. Demographically, accelerated accumulation of beta-amyloid in the brain tends to begin after the age of 50 years [11].

Subjective cognitive decline (SCD) refers to self-perceived decrement relative to one’s prior cognitive level [12], and awareness of such decline can be a risk for late-life dementia [13] even in the absence of performance deficiencies on objective cognitive tasks [14,15].

Berry fruit supplementation has the potential to produce varied health benefits, such as enhancement of metabolic function, moderation of inflammation and oxidative stress, improvement of vascular function, and augmentation of neuronal signaling [16]. Anthocyanins and proanthocyanidins are bioactive flavonoid compounds contained in blueberries. Anthocyanins impart the deep blue and purple color to blueberries. They are represented widely in fruits and vegetables although much more so in fruits [17,18]. Among the most common anthocyanins are the O-methylated peonidin, malvidin, and petunidin and the non-methylated cyanidin, delphinidin, and pelargonidin [17]. Cyanidin alone accounts for about 50% of anthocyanins present in plants [17]. Anthocyanins are involved in a variety of plant function, including coloration as a signal to pollinators and plant defense in the face of stressors such as UV radiation, oxidation, water and nutrient deprivation, and infection [19]. Some of these specific benefits appear to be translated to humans, especially under stress conditions [20] Proanthocyanidins also have plant defense functions, in particular antioxidant actions and response to stressors and infection [21], and have been implicated in risk reduction with respect to cancer, cardiovascular disease, metabolic disturbance, and neurocognitive disorders [21,22,23,24]. Notably, proanthocyanidins have been shown to reduce hyperglycemia in part via modulation of glucose transporter function [25] and inhibition of gluconeogenesis through 5′ adenosine monophosphate-activated protein kinase (AMPK) signaling [26].

In addition, there are indications that the composition of the gut microbiome influences insulin receptor sensitivity and other aspects of metabolic and immune function, effects that are mediated in part through increased cytokine expression [27]. Notably, gut microbiota can be altered favorably in response to nutritional changes, including consumption of bioactive compounds such as flavonoids. These effects have been associated with protection against disease conditions such as type 2 diabetes and cancer [28].

Generally, greater cognitive benefit has been observed in human supplementation studies involving older adults with mild cognitive impairment (MCI), a risk condition for dementia relative to studies with healthy older adults. Moreover, cognitive benefits with blueberry treatment in both animal and human studies have been observed in unimpaired younger individuals in the context of more challenging tasks, suggesting that blueberry intake can correct deficit and/or age-related cognitive decline and maintain function in the context of stress or greater challenge but does not produce supra-normal capability.

Improved cognitive performance with blueberry supplementation has been observed in aged animals [29,30,31], including enhancements of working memory, learning, and long-term memory [31,32,33,34,35,36]. Animal experiments also have shown anthocyanin accumulation in specific brain regions following blueberry consumption in association with enhanced cognitive performance [37,38] along with upregulation of neurotrophic factors [34,37,39].

Human trials have demonstrated improvements in long-term memory performance with blueberry supplementation in older adults with MCI [40] and in objective measures of executive ability and scales assessing perceived cognitive function in healthy older adults [41,42]. In addition, enhancement of cerebral perfusion and regional cerebral blood flow have been observed during cognitive task performance following several weeks’ blueberry supplementation [43,44].

Animal and clinical studies also indicate that blueberry consumption can improve metabolic function. Animal models of insulin resistance have been used to demonstrate improved insulin sensitivity and reduced glucose elevation with blueberry supplementation [45]. Similarly, in models of obesity, blueberry treatment improved insulin and glucose control [46,47,48,49]. In human population studies, blueberry consumption has been associated with reduced risk of type 2 diabetes [50] and with mitigation of insulin resistance and inflammation as measured by C-reactive protein [51]. In controlled trials involving overweight, insulin-resistant individuals, blueberry supplementation was associated with improved insulin sensitivity [52], and in a small crossover study involving healthy young adults, the inclusion of blueberry in a meal was shown to extend the acute postprandial glucose response relative to a matched control condition [53]. In another crossover study with repeated cognitive and metabolic assessments following a high-carbohydrate meal, healthy middle-aged, blueberry-treated participants maintained performances on challenging executive tasks and exhibited relatively lower levels of post-meal glucose and insulin [54].

Diminished brain energy utilization, evident as a reduction in the cerebral metabolic rate of glucose, is characteristic of AD [55] and is apparent in the brains of younger individuals with increased risk for dementia well before cognitive decline becomes apparent [56]. Flavonoid compounds also can influence bioenergetic function. Flavanols and anthocyanins induce phosphorylation of endothelial nitric oxide (eNO) synthase, leading to increased production of eNO and improved vascular function [57]. Further, NO modulates mitochondrial reactive oxygen species through upregulation of PGC-1α (peroxisome proliferator-activation receptor gamma coactivator-alpha), a regulator of mitochondrial function and biogenesis [58,59]. Notably, a recent human supplementation trial showed that cognitive benefits were associated with ongoing intake of the unmodified food form of blueberry anthocyanins but not with anthocyanin metabolites [60].

This manuscript reports on a human trial designed to provide new data regarding the efficacy of blueberry supplementation for improving cognitive performance in middle-aged men and women with greater risk for age-related cognitive decline and dementia determined by the presence of early metabolic disturbance and subjective cognitive decline. Demonstrating benefit in this preliminary study would provide support for further investigation of the potential of blueberry consumption as a preventive strategy implemented in midlife against cognitive decline.

## 2. Materials and Methods

### 2.1. Study Design

This was a randomized, double-blind, placebo-controlled trial evaluating the effect of blueberry supplementation on cognitive performance in non-diabetic, middle-aged, overweight men and women with subjective cognitive decline. Participants were recruited from the community in the region in and around Cincinnati, OH, USA. Flyers and email notices distributed to academic health center employees were used to solicit participation of individuals aged 50 to 65 years who had gained weight in midlife and who were aware of having developed cognitive inefficiency.

**Figure 1:**
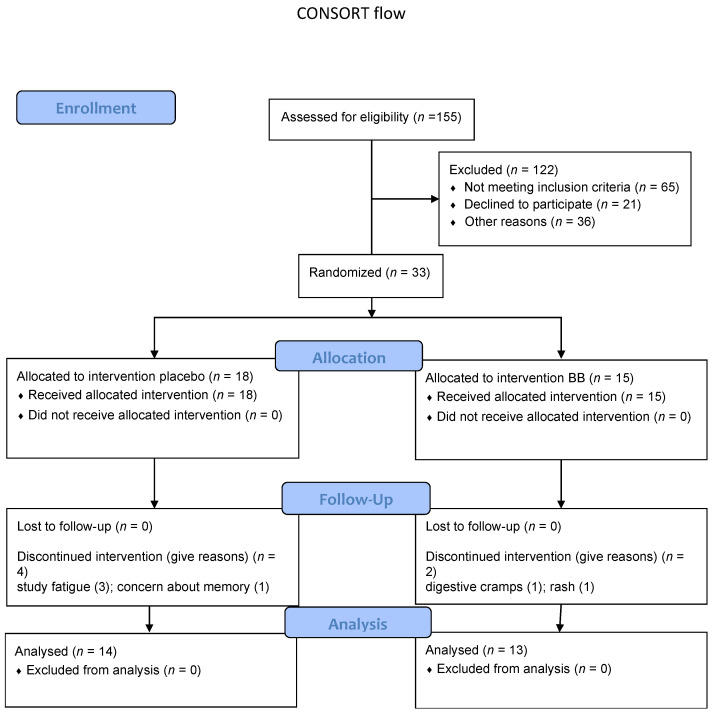
CONSORT flow diagram showing data concerning participant enrollment, randomized group allocation, completion information, and number of participants included in the group analyses. BB = blueberry treated group.

The study protocol was approved by the University of Cincinnati Medical Institutional Review Board and registered with Clinical Trials Identifier NCT02751866. Each participant reviewed and signed the informed consent document. Major assessments were performed prior to and after the 12-week intervention and included neuropsychological and mood measures, blood samples for determination of metabolic and lipid markers, and anthropometric indices. Diet diary records were completed by participants during the week prior to enrollment and during the week prior to the final study visit to assess change in anthocyanin consumption in the background diet. We also included measures of peripheral platelet mitochondrial function as exploratory markers of potential change in bioenergetic function associated with blueberry supplementation [61]. Figure 1 contains data on participant screening, enrollment, and retention.

### 2.2. Inclusion Criteria

(1) Men and women 50 to 65 years old;
(2) Body mass index (BMI) = 25 or greater;
(3) Subjective cognitive complaints reflecting awareness of decline in cognitive capability from a prior level;
(4) Ability to comprehend and comply with the research protocol;
(5) Provision of written informed consent.

### 2.3. Exclusion Criteria

(1) Diagnosis of neurological condition or neurocognitive disorder, such as mild cognitive impairment, Alzheimer’s disease, or Parkinson’s disease;
(2) Current or past psychiatric condition, such as psychosis or major mood disorder, causing a persisting change in level of occupational or social functioning;
(3) Current or past substance use causing physiological dependence or change in functional capability;
(4) Diabetes or kidney or liver disease;
(5) Regular use of medication or dietary supplement that might affect outcome measures, such as benzodiazepines, psychostimulants, and berry fruit extracts.

### 2.4. Telephone Screening

Each prospective participant completed an initial phone interview during which we provided an overview of the requirements for study participation and reviewed inclusion and exclusion criteria. The Academic and Medical History Questionnaire [62] was used to obtain demographic and educational information; information concerning medical, neurological, and psychiatric conditions; and information concerning use of substances, medications, and supplements. We also administered the Everyday Memory Questionnaire [63] to characterize the nature and extent of perceived memory decline.

### 2.5. Enrollment and Final Study Visits

Individuals who qualified were assigned randomly to either daily blueberry powder (BB) or placebo powder (PL) supplementation. We performed neuropsychological studies, measured anthropometric parameters and collected fasting blood samples at the enrollment and final study visits. Three-day diet diaries completed during the prior week also were collected at these study visits. A 6-week supply of supplement packets was given to participants at the enrollment visit.

### 2.6. Interim Visit

Participants returned during week 6 of the intervention for a brief visit. Unused supplement packets were collected, and we supplied supplement packets for the final 6-week period and diet record forms to be completed during the week before the final visit.

### 2.7. Whole, Freeze-Dried Blueberry and Placebo Powder and Supplementation Regimen

Those who qualified for participation were asked to abstain from berry fruit consumption for 14 days before study enrollment and for the term of the intervention. Excluded foods included all berry fruits, juices, and extracts. We did not attempt to control anthocyanin consumption from non-berry fruits and vegetables, reasoning that this would be burdensome and not representative of typical consumption habits for free living individuals.

We utilized blueberry and placebo powders supplied by the U.S. Highbush Blueberry Council, Folsom, CA, USA. Extrapolations from quantities used in animal and past human experiments indicated that daily dosing with 0.5 to 1.0 c whole-fruit equivalent had been effective in producing biological and behavioral responses [45]. Given the relatively extended term of the intervention and to reduce participant burden, we used a daily dosage of 0.5 c whole-fruit equivalent administered once each day. The placebo powder was matched for sugars, glycemic load, appearance, and taste but did not contain fiber. The blueberry and placebo powders were packaged in individual dose packets, each marked with a code to preserve the double blinding. We asked participants to consume the contents of one packet with either the morning or evening meal and recommended that the powder be mixed with water. Participants were asked to return both emptied and unused packets at the interim and final study visits.

### 2.8. Neuropsychological Assessment

The neurocognitive protocol included measures of executive abilities (inhibitory control, task switching, and lexical access) and long-term memory, cognitive domains implicated in aging and late-life dementia. Equivalent, alternate forms of the long-term memory tasks were used to minimize practice effects associated with repeated exposure to specific test items. We also administered a scale to characterize participants’ perceptions of memory difficulties in daily life activities and a mood measure as a potential covariate for the cognitive outcomes. Table 1 shows the neuropsychological tests and associated cognitive domains.

#### 2.8.1. Controlled Oral Word Production

This task involves rapid oral word production. It depends on integrity of lexical access and executive control processes, such as updating, shifting mental set, and inhibitory control [67], to retrieve particular classes of words from one’s lexical knowledge store. The task involves timed, oral word production trials involving phonemic and category constraints. It yields two outcome measures: the number of words produced that begin with specified letters of the alphabet and the number of words produced that are representative of a particular semantic category [68].

#### 2.8.2. The California Verbal Learning Test, Second Edition

The California Verbal Learning Test, Second Edition [64] is among the better-designed list learning procedures for assessment of learning and long-term memory function. It yields measures of learning efficacy, delayed recall, and interference in memory. Equivalent alternate forms are available for repeated measurements in research contexts.

#### 2.8.3. Verbal Paired Associate Learning

This associative learning task has been used in standardization studies [65] and in prior berry trials [40]. It was included in addition to the list learning procedure because it involves more challenging and resource-intensive memory encoding. Alternate forms were used at the enrollment and final assessments.

#### 2.8.4. The Everyday Memory Questionnaire

The Everyday Memory Questionnaire (EMQ) [63] was used to assess self-reported memory problems in day-to-day activities. This instrument was developed to complement objective memory assessments and has demonstrated reliability and sensitivity [51] and was included to provide a systematic means of surveying the nature of memory difficulties in the context of subjective cognitive decline. The EMQ yields a total score and three factor scores including a memory retrieval factor (word-finding difficulty and problems recalling recent events), an attentional tracking factor (losing track of train of thought and intentions), and a third factor assessing forgetfulness reflecting poor memory encoding, such as misplacing possessions and reading without awareness of prior exposure to the material.

#### 2.8.5. The Beck Depression Inventory II

The Beck Depression Inventory-II [66] was used to quantify the level of depressive symptoms such as hopelessness, irritability, and guilt. It provides a measure of the intensity of such symptoms and is suitable for monitoring change in mood over time.

### 2.9. Metabolic Measures

Fasting blood samples were obtained, pre-processed, and stored at −80 °C until delivery in batches to the University of Cincinnati Metabolic Diseases Institute for serum glucose and insulin assays and determination of lipid values, including total cholesterol, high-density lipoprotein, low-density lipoprotein, and triglycerides. Whole blood was refrigerated and then transported to the Clinical Translational Research Center where glycated hemoglobin determinations were performed.

### 2.10. Anthropometric Measures

We measured height and body weight for calculation of BMI and waist circumference at the narrowest waist at the enrollment visit. Body weight and waist circumference were measured again during the final study visit.

### 2.11. Mitochondrial Oxygen-Consumption Rate

We assessed change in mitochondrial function in peripheral platelets. Extent of uncoupling and state III oxygen-consumption rate were assessed using the Seahorse XF24 Extracellular Flux Analyzer [61]. Whole blood was centrifuged, and platelet-rich plasma harvested. Following the measurement of basal respiration, mitochondria were stimulated by the addition of pyruvate, malate, and succinate. Oligomycin was used to inhibit ATP synthase, reducing electron flow and oxygen consumption. Respiration was uncoupled from phosphorylation using p-trifluoromethoxy carbonyl cyanide phenyl hydrazone (FCCP), allowing for the measurement of maximum respiration. The mean of at least three individual wells from the same sample was calculated for each patient. From these values, state III respiration (malate/pyruvate/succinate over basal respiration) and percent maximal uncoupling (oligomycin over FCCP) were calculated.

### 2.12. Diet Diary Records

Participants were instructed to record their intake of food and beverages for three consecutive days, including one weekend day and two weekdays, during the week before enrollment and during the week before the final study visit to monitor nutritional intake external to the study. The Nutrition Data Systems for Research (NDSR; Nutrition Coordinating Center, University of Minnesota, Minneapolis, MN, USA) was used to tabulate total energy and macronutrient intake, and a dedicated module for dietary polyphenol intake was used to compile anthocyanin consumption. These data were used to quantify anthocyanin intake outside the research from in the background diet during the period of the intervention to assess the possibility of between-group differences in consumption of anthocyanins external to the study. It also allowed a rough estimate concerning the effectiveness of the prescription against berry intake.

### 2.13. Statistical Analyses and Power

Analysis of covariance (ANCOVA) tests were performed for each cognitive domain and for the metabolic and anthropometric measures. These analyses tested between-group differences in final visit outcome scores while standardizing the parallel scores obtained at enrollment so that the effect of the intervention on the measures was isolated [69]. We calculated Cohen’s *f* statistic to determine effect size estimates for significant effects and trends. Cohen’s *f* is an extension of the effect size statistic (*d*) for application in *F*-tests, and values are characterized as small (0.10), medium (0.25), and large (0.40) [70]. In this study, Cohen’s *f* values ranged from medium to large. Given the sample size and alpha probability = 0.05, the calculated power was greater than 0.80 [70]. We also performed regression analyses to evaluate the association of change in metabolic and anthropometric factors during the intervention with the significant outcome measures. In these analyses, the final visit outcome score was the dependent measure, and the corresponding enrollment visit score along with the metabolic or anthropometric change score (final visit value minus baseline visit value) served as covariate measures.

## 3. Results

Thirty-three participants were enrolled. Six participants (18%) were unavailable for final visit assessments.

Table 2 contains demographic, anthropometric, metabolic, mood, and cognitive characteristics of the sample at the time of enrollment. There was no difference between the groups for any of these factors. By design, the sample exhibited elevated BMI and waist circumference values, both of which are consistent with the presence of insulin resistance [71,72]. For both groups, mean fasting insulin values were in the range of mild to moderate hyperinsulinemia [6,71,72], and mean fasting glucose values were below the threshold for type 2 diabetes [71]. Mean glycated hemoglobin values were moderately elevated but below the diabetic range [71]. The level of depressive symptoms was low in each group, and there was no group difference in the EMQ total score representing perceived memory difficulties in everyday activities.

**Figure 2:**
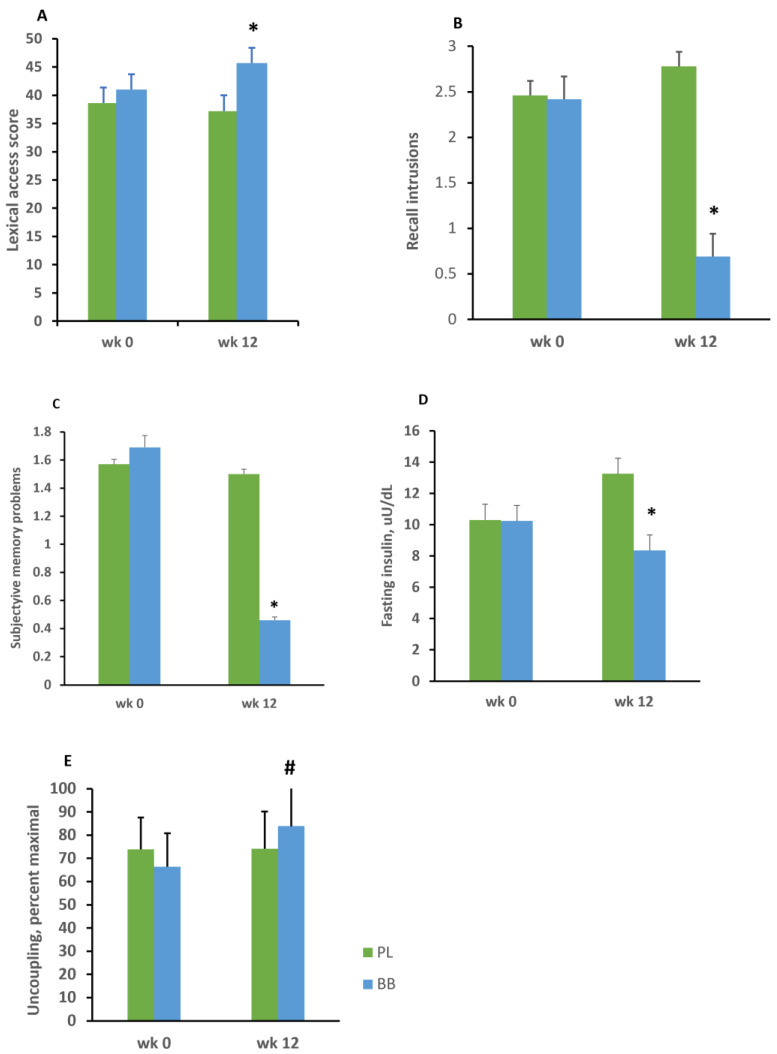
(**A**). Lexical access performance by group at enrollment and at the end of the intervention, as measured by the letter form of the Controlled Oral Word Association task. At week 12, the BB group showed improved performance, * *F*(1,24) = 10.67, *p* = 0.003, Cohen’s *f* effect size = 0.66. (**B**). Recall intrusion errors by group on the California Verbal Learning Test. The BB group exhibited fewer intrusion errors after 12 weeks supplementation, * *F*(1,24) = 4.69, *p* = 0.04, Cohen’s *f* effect size = 0.20. (**C**). Perceived day-to-day memory difficulties by group as measured by the Everyday Memory Questionnaire, factor 3. The BB group reported a reduction of forgetfulness and encoding-type failures after 12 weeks’ supplementation, * *F*(1,24) = 4.93, *p* = 0.03, Cohen’s *f* effect size = 0.45. (**D**). Mean fasting insulin values at enrollment and after 12 weeks by group. The BB group showed a reduction of insulin following supplementation, * *F*(1,24) = 4.62, *p* = 0.04, Cohen’s *f* effect size = 0.44. (**E**). Trend for enhanced mitochondrial uncoupling in peripheral platelets for blueberry-treated participants, # *F*(1,14) = 2.17, *p* = 0.11. Error bars = SEM. PL = placebo group. BB = blueberry group. Wk 0 = pre-intervention assessment. Wk 12 = assessment during the final week of the intervention.

We observed comparatively improved lexical access for the BB group specifically for phonemic access as shown in Figure 2, panel A. This effect was robust, *F*(1,24) = 10.67, *p* = 0.003, with a large effect size, Cohen’s *f* = 0.66. On the other hand, there was no effect for the category form of the COWA, *F*(1,24) = 0.33, *p* = 0.56.

There was no between group effect for the Verbal Paired Associate Learning Test, *p* = 0.28. Additionally, measures of learning rate, delayed recall, and recognition memory derived from the California Verbal Learning Test were not affected by the intervention. However, we did observe a statistically significant reduction of recall intrusion errors for the BB group, *F*(1,24) = 4.69, *p* = 0.04, Cohen’s *f* = 0.20 (Figure 2B).

We analyzed between group effects for the Everyday Memory Questionnaire total score and for each of the three factors. There was a trend indicating fewer memory complaints overall for the BB group, EMQ total score, *p* = 0.10, Cohen’s *f* = 0.34. This trend was based largely on the significant effect for factor 3 of the EMQ, which assesses forgetfulness and memory encoding difficulties, *F*(1,24) = 4.93, *p* = 0.03, Cohen’s *f* = 0.45 (Figure 2C).

We observed significant decline in fasting insulin at week 12 for the BB group relative to the PL group, *F*(1,24) = 4.62, *p* = 0.04, Cohen’s *f* = 0.45 (Figure 2D). This reduction in fasting insulin occurred in the absence of a between group difference in total daily energy intake, *p* = 0.99, or daily consumption of carbohydrate, *p* = 0.66; protein, *p* = 0.65; and fat, *p* = 0.16. The change in insulin within the BB group was substantial, with a reduction to 8.3 µU/mL evident at week 12. There also was a strong trend indicating that change in body weight during the trial predicted fasting insulin values, standardized beta (*ꞵstd*) = 0.21, *p* = 0.07. There was no effect of the intervention on lipid values, including total cholesterol, *p* = 0.45; high-density lipoprotein, *p* = 0.68; low-density lipoprotein, *p* = 0.30; and triglycerides, *p* = 0.74. In addition, there was no between-group difference for any anthropometric measure, including BMI, *p* = 0.24; body weight, *p* = 0.87; and waist circumference, *p* = 0.85, or for any other metabolic parameter, including fasting glucose, *p* = 0.28; glycated hemoglobin, *p* = 0.26; and calculated insulin resistance (HOMA2 IR), *p* = 0.17.

**Figure 3:**
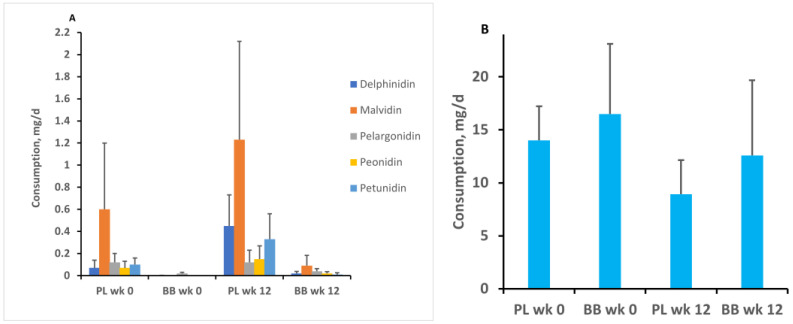
Mean daily consumption external to the study for each of the major anthocyanins by group at enrollment and after 12 weeks. There was no between-group difference in consumption and no change relative to pre-intervention levels. Panel (**A**) shows consumption of delphinidin, malvidin, pelargonidin, peonidin, and petunidin, which were consumed at relatively lower levels. Panel (**B**) represents cyanidin levels, for which there was considerably greater intake. Note the difference in range of values on the vertical axes between panel (**A**) and panel (**B**). Error bars = SEM. PL = placebo group. BB = blueberry group. Wk 0 = pre-intervention assessment. Wk 12 = assessment during the final week of the intervention.

We obtained three-day diet records completed by participants during the week before enrollment and during week 12 of the intervention in order to measure anthocyanin intake external to the study. Consumption of each of the six major anthocyanins was tabulated, and we performed repeated measures ANOVA to assess between-group change in intake for each anthocyanin during the intervention. These analyses indicated that there was no change in background consumption of any anthocyanin during the intervention. However, it was evident that for both groups, cyanidin intake in the background diet was higher than intake of delphinidin, malvidin, pelargonidin, peonidin, and petunidin (Figure 3).

The intervention had no effect on mood symptoms as measured by the Beck Depression Inventory. Symptom levels were low at enrollment and did not change appreciably for either group during the study, *F*(1,24) = 0.39, *p* = 0.53.

The dataset for the mitochondrial studies was limited because some samples were lost during transport and because of difficulty harvesting sufficient platelet quantities from some of the samples. Data were available for 17 participants, including 10 from the placebo group and 7 from the BB group. There was a modest trend suggesting enhanced uncoupling for the BB group (74.1 vs. 83.8), *F*(1,14) = 2.17, *p* = 0.11, Cohen’s *f* = 0.44 (Figure 2E). There also was a seemingly large difference between the groups for state III respiration (151.4 vs. 176.1). However, this was not confirmed statistically, *F*(1,14) = 0.28, *p* = 0.50.

## 4. Discussion

We found that daily blueberry powder supplementation equivalent to 0.5 c whole fruit for 12 weeks improved performance on a lexical access task and reduced interference of extraneous information in memory. These cognitive performance enhancements can be understood as reflecting improved executive control among the blueberry-treated participants [67]. The findings are a demonstration that blueberry supplementation can produce measurable cognitive benefit in the context of aging and insulin resistance.

The effect for improved lexical access for the blueberry group was observed solely for phonemic (or letter) production, and there was no effect for production under the category constraint. This specificity is interesting. Each form of the Controlled Oral Word Production task depends on executive abilities, such as updating and inhibitory control. Letter fluency has been shown to involve relatively greater executive challenge, as it requires greater inhibition to suppress competing, incorrect phonemically similar words [73], for example, suppressing the term “physics” as a response despite the phonemic similarity to other acceptable words that begin with the letter “f”. Such intrusion errors resulting from failure of inhibitory constraint have been described in other contexts as resulting from inefficiency suppressing irrelevant or non-target information in working memory [74].

We also observed fewer intrusion errors for the blueberry-treated group on the California Verbal Learning Test despite the absence of differences for measures of learning, delayed recall, and recognition memory. This selective enhancement involved a reduction of errors representing intrusion of non-target or extraneous items during learning trials, again reflecting improved inhibitory control in the suppression of such intrusions. This sort of interference in learning and memory also has been observed in healthy older adults [75] and in patients with frontal lesions who exhibit impairment on other executive tasks [76,77]. Accordingly, the benefit associated with blueberry supplementation with respect to reduced interference was evident in an aspect of learning that relies on executive control processes for encoding and suppression of extraneous material.

More generally, overweight middle-aged individuals have been shown to exhibit executive impairment. Obesity has been associated with changes in cerebral white matter fibers specifically in the genu of the corpus callosum, a structure that connects frontal lobe regions mediating executive ability [78]. This is consistent with the notion that relatively greater executive difficulty would be present in our overweight middle-aged sample. The specific effect for phonemic word production observed in this study contrasts with converse findings obtained in a prior trial with older participants diagnosed with mild cognitive impairment [60]. Individuals with MCI and Alzheimer’s disease characteristically exhibit impairment of semantic access that reflects a more generalized, progressive disorder of conceptualization [79]. In that prior study with older MCI participants [60], we found enhanced performance among the blueberry-treated participants on the category form of the COWA task, presumably because of the relatively greater deficit of semantic access in that sample. Such complementary findings in samples representing different demographic and cognitive characteristics would seem to reflect the fact that blueberry supplementation can enhance cognitive performance in the context of impairment or greater challenge but not necessarily when applied in the absence of cognitive decrement.

In the overweight, middle-aged sample involved in this study, cognitive enhancement reflected improvement on tasks that depend on executive control. From a developmental perspective, executive ability tends to decline beginning in midlife, even in the context of nonpathological aging [80]. In addition, there is evidence that this effect is magnified in individuals who are overweight and have metabolic disturbance regardless of age [81,82,83]. Further, executive impairment associated with excess body weight is most apparent on tasks assessing inhibitory control and working memory [84]. On the other hand, learning and long-term memory per se tend to decline in old age [85]. Consistent with these notions, there was no effect of blueberry supplementation on memory measures in this middle-aged sample, while we have shown previously that blueberry supplementation improves memory performance in older adults with MCI [40].

The improvement of executive ability observed with objective measures also was reflected in the experience of fewer difficulties for the blueberry group documented in lower scores on the Everyday Memory Questionnaire. This effect was evident specifically for factor 3 of the EMQ, which measures memory difficulties resulting from ineffective memory encoding and poor source memory, which are problems that are associated with executive impairment [86]. These data concerning day-to-day difficulties are useful, as they corroborate the selective findings obtained with the objective cognitive measures. Furthermore, the perception of improvement in everyday functioning among those in the blueberry-treated group might be considered particularly meaningful in the context of a participant sample composed of individuals with subjective cognitive decline, that is, improvement with respect to a risk factor for future dementia.

The other major finding was that blueberry supplementation produced a reduction of fasting insulin. The insulin reduction occurred in the absence of change in glucose or glycated hemoglobin. These observations recapitulate other animal and human reports [45,46,52] indicating that blueberry anthocyanins can reduce endogenous insulin demand without increasing glycemia in the context of obesity and insulin resistance, which is the case in our participant sample. Several mechanisms have been investigated in this regard [87], such as activation of peroxisome proliferator-activated receptors (PPARs) [46,88,89] and suppression of carbohydrate metabolism in the gut [90,91]. Notably, insulin values greater than 8.3 µU/mL are associated with suppression of lipolysis [92] so that the observed reduction of insulin from 10.2 µU/mL to 8.3 µU/mL in the blueberry group is a clinically meaningful correction of hyperinsulinemia. Further, the reduction of insulin may have been reflected in the association of change in body weight with insulin. Anthocyanin consumption has been associated with anti-obesity effects that appear to be mediated through multiple mechanisms. These include activation of AMPK; downstream suppression of fatty acid synthase; an agonistic effect on PPAR-α, which increases fatty acid oxidation and ketone production [93]; and suppression of PPAR-ɤ to mitigate triglyceride storage [93,94]. The activation of AMPK also is associated with activation of peroxisome proliferator-activated receptor-gamma coactivator (PCG)-1α and with increased mitochondrial biogenesis [94]. Reduction of insulin also would be expected to contribute to cognitive benefit given the strong association of insulin resistance and associated hyperinsulinemia with neurocognitive decline and progression of neuropathology [95,96].

Finally, we observed a trend suggesting elevated mitochondrial uncoupling in the blueberry-treated group. The reduced number of available mitochondrial assays may have been the basis for the weaker statistical result for this measure. Flavonoids can be protective against mitochondrial dysfunction and related oxidative stress [97], and anthocyanin compounds can influence mitochondrial respiration. In particular, mild or partial uncoupling has been associated with the actions of certain anthocyanins [98] and with reduced oxidative stress, preservation of tissue function with aging, and with extended lifespan [99]. However, in the context of insulin resistance, uncoupling may worsen dysfunction [99].

Our data concerning anthocyanin consumption in the background diet are consistent with the notion that participants in both the placebo and blueberry groups maintained the research prohibition against consuming berry fruits during the intervention. Further, there was no difference in anthocyanin intake between the groups. Presumably, the relatively greater intake of cyanidin observed in both groups was due to its representation in a large variety of non-berry fruits and vegetables [17].

The relatively small sample size is a study limitation although our observation of cognitive benefit is consistent with other studies indicating such enhancements associated with short- to moderate-term blueberry supplementation. In addition, effect size estimates were moderate to large. This trial represents a first effort aimed at supplementation in a sample of middle-aged individuals selected on the basis of elevated risk for future health problems and late-life dementia due to their metabolic condition and the presence of subjective cognitive decline. In this regard, it provides novel data concerning the potential of blueberry supplementation as a preventive intervention. The inclusion criterion of BMI > 25 and exclusion of those with diagnosed diabetes were effective in obtaining a sample of individuals with insulin resistance.

## 5. Conclusions

In summary, this study demonstrated that blueberry supplementation has neurocognitive benefit in middle-aged individuals with insulin resistance and elevated risk for future dementia. We observed selective improvement of executive abilities consistent with the notion that ongoing blueberry intake can mitigate deficiencies or deficits, as has been observed numerous times in prior animal and clinical studies. The findings suggest that supplementation has potential for protection against future neurocognitive decline in vulnerable individuals. Further, the level of blueberry supplementation employed in animal studies typically has not been greater than that employed in the current human trial and would be feasible for most individuals [45]. In addition, we obtained data concerning mechanistic factors including correction of hyperinsulinemia and possibly benefits for mitochondrial function. Future research assessing blueberry supplementation over longer periods with longitudinal cognitive assessments would be valuable not only to assess its influence on progression of cognitive decline but also to investigate further the mechanisms of neurocognitive benefit. The role of blueberry anthocyanins and proanthocyanidins in the enhancement of metabolic and mitochondrial function seems particularly salient, as these factors are intrinsic to neurodegenerative processes.

## Figures and Tables

**Table 1:** Neuropsychological measures and respective cognitive domains.

Neurocognitive Measure	Cognitive Domain
Controlled Oral Word Association Test [64,65]	Executive ability
California Verbal Learning Test [64]	Learning/memory; Executive ability
Verbal Paired Associate Learning test [65]	Learning/memory
Everyday Memory Questionnaire [63]	Self-rated memory function
Beck Depression Inventory [66]	Mood

**Table 2:** Sample characteristics at enrollment by group.

Factor	Placebo (*n* = 14)	Blueberry (*n* = 13)	*t*-Value	*p*
Age, years	57.2	55.6	1.01	0.32
Education, years	14.8	16.3	1.70	0.10
Body weight, kg	94.2	93.0	0.15	0.87
BMI	33.2	31.7	0.62	0.53
Waist circumference, cm	107.3	106.7	0.10	0.91
Fasting insulin, µU/mL	10.3	10.2	0.01	0.98
Fasting glucose, mg/dL	109.5	99.3	0.64	0.52
HbA1c, %	6.16	5.67	0.89	0.38
Total cholesterol, mg/dL	197.6	200.8	0.66	0.51
HDL, mg/dL	53.3	61.0	1.34	0.18
LDL, mg/dL	116.1	128.6	1.00	0.32
Triglycerides, mg/dL	140.9	98.4	1.53	0.13
BDI	6.9	9.2	0.99	0.32
EMQ	20.7	15.4	1.53	0.13

Note. BMI = body mass index. HbA1c = glycated hemoglobin. HDL = high-density lipoprotein. LDL = low-density lipoprotein. BDI = Beck Depression Inventory. EMQ = Everyday Memory Questionnaire.

## Data Availability

Data will be made available when the manuscript is accepted for publication.
